# Convolutional neural network-based respiration analysis of electrical activities of the diaphragm

**DOI:** 10.1038/s41598-022-21165-9

**Published:** 2022-10-05

**Authors:** Hyun-Gyu Lee, Gahee Lee, Juyoung Lee

**Affiliations:** 1 Department of Medical Education and Medical Humanities, College of Medicine, Inha University, Incheon, South Korea; 2 Department of Pediatrics, College of Medicine, Inha University, Incheon, South Korea

**Keywords:** Biotechnology, Computational biology and bioinformatics, Health care, Medical research

## Abstract

The electrical activity of the diaphragm (Edi) is considered a new respiratory vital sign for monitoring breathing patterns and efforts during ventilator care. However, the Edi signal contains irregular noise from complex causes, which makes reliable breathing analysis difficult. Deep learning was implemented to accurately detect the Edi signal peaks and analyze actual neural breathing in premature infants. Edi signals were collected from 17 premature infants born before gestational age less than 32 weeks, who received ventilatory support with a non-invasive neurally adjusted ventilatory assist. First, a local maximal detection method that over-detects candidate Edi peaks was used. Subsequently, a convolutional neural network-based deep learning was implemented to classify candidates into final Edi peaks. Our approach showed superior performance in all aspects of respiratory Edi peak detection and neural breathing analysis compared with the currently used recording technique in the ventilator. The method obtained a f1-score of 0.956 for the Edi peak detection performance and ${R}^{2}$ value of 0.823 for respiratory rates based on the number of Edi peaks. The proposed technique can achieve a more reliable analysis of Edi signals, including evaluation of the respiration rate in premature infants.

## Introduction

Respiratory monitoring is essential during ventilator care in premature infants with respiratory distress. During invasive ventilation through the endotracheal tube, a pneumotachograph is the standard method for monitoring respiration^1^. However, because it is easily affected by leakages, it is not designable for a premature infant who uses an uncuffed tube, and during non-invasive ventilation^1^. Thermistors or hot-wire anemometers that use temperature sensing are also highly affected by the humidity of the ventilator or leakages^1^. The most commonly used method for monitoring respiration during non-invasive ventilation is chest impedance; however, it is prone to motion artifacts^2^. To overcome the limitation of chest impedance, a surface diaphragmatic electromyogram (EMG) has been proposed in neonates^3^. However, a similar susceptibility of surface EMG to motion artifacts, as in case of chest impedance, has been reported^3^. In addition, EMG as well as chest impedance rely on surface skin electrodes, which may harm the fragile skin of preterm infants.

**Figure 1:**
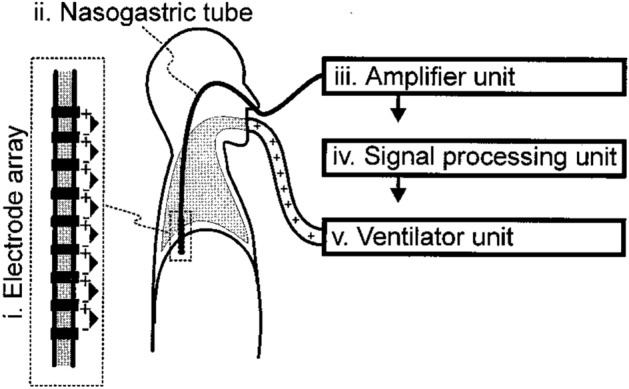
Description of the setup for measurement of the electrical activity of the diaphragm. Electrode array arrangement (i) attached to a nasogastric tube (ii) normally used for feeding or other purposes. The electrode array is positioned in the esophagus at the level of end perpendicular to the crural diaphragm such that the active muscle creates an electrically active region around the electrode. Signals from each electrode pair on the array are differentially amplified (iii) and digitalized into a personal computer and filtered (iv) to minimize the influence of cardiac electrical activity, electrode motion artifacts, and common noise, as well as other sources of electrical interference. The processed signal’s intensity value is displayed for monitoring purposes or fed to the ventilator (v) to control the timing and/or levels of the ventilator assist. Reprinted by permission from Springer Nature^5^.

Neurally adjusted ventilatory assist (NAVA) is a ventilatory support mode, in which the electrical activity of the diaphragm (Edi) is used to control the breathing cycle and support magnitude^4^. Edi is the most ideal signal that can be recorded noninvasively to represent the neural respiratory drive originating in the respiratory centers^5^. Edi is measured with an array of tiny sensors on the patient’s feeding tube (Edi catheter) at the level of the gastroesophageal junction^6^. Sensors in this position pick up the Edi signals from the crural diaphragm, which forms a scarf-like structure around the lower esophageal sphincter. An array of sensors automatically tracks the diaphragm contraction during spontaneous breathing^5^. After recorded crural diaphragm EMG signals are transformed to the Edi signal through filtering, processing and amplification, this signal is output to a ventilator (Fig. 1), which then delivers respiratory assistance in proportion to the peak Edi value^5^. Because the Edi signal provides real-time information about breathing patterns and efforts^6–9^, obtaining an accurate recording of Edi is important for monitoring unstable patients. In addition, because respiratory support with NAVA mode depends on the Edi signal, getting a reliable Edi signal is crucial for operating NAVA.

However, it is challenging to obtain precise Edi signals that are sufficiently free of artifacts, especially in premature infants, owing to electrode motion artifacts, esophageal peristalsis, and other bioelectric noises. To reduce noise in the Edi signal, the electrode array in the Edi catheter was determined concerning the position of the diaphragm muscle fiber^10^, and the Edi signals were processed using the double subtraction technique^11^. However, studies on Edi signal quality have been conducted only in healthy adults, and no data have proven whether noises in the Edi signal are acceptable in premature infants. Moreover, although neonates and infants vary widely in their lung volume, tidal volume, and diaphragm thickness, the inter-electrode distance of the Edi catheter is universally 6 mm for all infants (whether they are 0.5 kg or 5.0 kg) ^10^. In fact, more noise is noted in the Edi signal as the infants are premature, resulting in increased variability in respiratory parameters in the neonatal intensive care unit^12^.

Owing to the high variability of the Edi signal noise, it is challenging to properly analyze respiration using the conventional method. The ServoTracker (Maquet Critical Care, Solna, Sweden) was used to analyze respiration from the Servo ventilator (Getinge, Gothenburg, Sweden), which detects many false Edi peaks regardless of respiration. Therefore, in this study, we aimed to precisely detect peaks in the Edi signal using signal processing and a convolutional neural network (CNN) to analyze the actual neural breathing of premature infants.

## Methods

### Data collection and study design

We prospectively collected Edi signals from premature infants born before gestational age < 32 weeks and received ventilatory support with non-invasive NAVA. The institutional review board of Inha University Hospital of Korea approved the study protocol (IRB No: INHAUH 2021-04-034), and written informed consent was obtained from the parents before data collection. All the study methods were performed in accordance with the Declaration of Helsinki (as revised in 2013).

One hour of Edi signals from each infant was acquired from the Servo-n ventilator through an RS232 interface at a sampling rate of 100 Hz. These data were simultaneously recorded using the dedicated software ServoTracker. The recorded log files were extracted to separate files to obtain all raw pressure, flow, and Edi data. Two independent neonatologists performed breath-by-breath analyses of simultaneous pressure, flow, and Edi curves using customized software based on Microsoft Excel (Microsoft, Redmond, Washington, USA)^13^. The Edi peaks determined through breath-by-breath analyses were defined as the ground truth.

**Figure 2:**
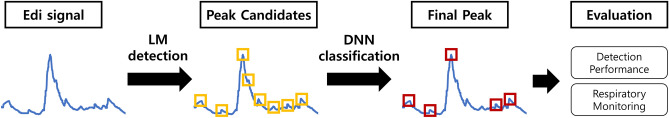
Overview of the respiration analysis algorithm with Edi peak detection.

We proposed a peak detection method for the respiration analysis of Edi signals. In this study, our peak detection method consisted of candidate peak detection using a local maximum (LM) and peak classification using a CNN-based deep neural network (DNN), as shown in Fig. 2. First, the positions of the maximum Edi value in the local signal with a fixed length were determined. Subsequently, LM detection found as many candidate peaks as possible without missing the true peaks, and the final peaks were selected from among the candidate peaks using the DNN classifier. Finally, the performance of our method for Edi peak detection was evaluated and synchrony analyses related to respiration was compared.

### Edi peak detection

In this study, as many candidate Edi peaks as possible were identified, including pseudo-peaks. The LM for the candidate peak is defined as follows:

1 $$LM\left(x\right)=\left\{\begin{array}{l}1,\quad if\;max\;{Edi}_{a, b}=Edi(x), s.t. x\in \left[a,b\right], x=\frac{a+b}{2},\\ 0, \quad others\end{array}\right.$$

where, $x$ is a timestamp (microseconds, µs) of the Edi signal, ${Edi}_{a, b}$ is the Edi signal between timestamps $a$ and $b$, and $Edi(x)$ is the Edi value at timestamp $x$. If the $LM\left(x\right)$ output is 1, the timestamp $x$ is considered the local maximum location. If $Edi(x)$ equals $\mathit{max}{Edi}_{a, b}$ under the constraints, $Edi(x)$ has the highest value in ${Edi}_{a, b}$ with $x$, which is in the middle position between $a$ and $b$. The difference between $a$ and $b$ is the length ${\Omega }_{\mathrm{LM}}$ of the signal. More peaks are detected when the interval length ${\Omega }_{\mathrm{LM}}$ is smaller.

**Figure 3:**
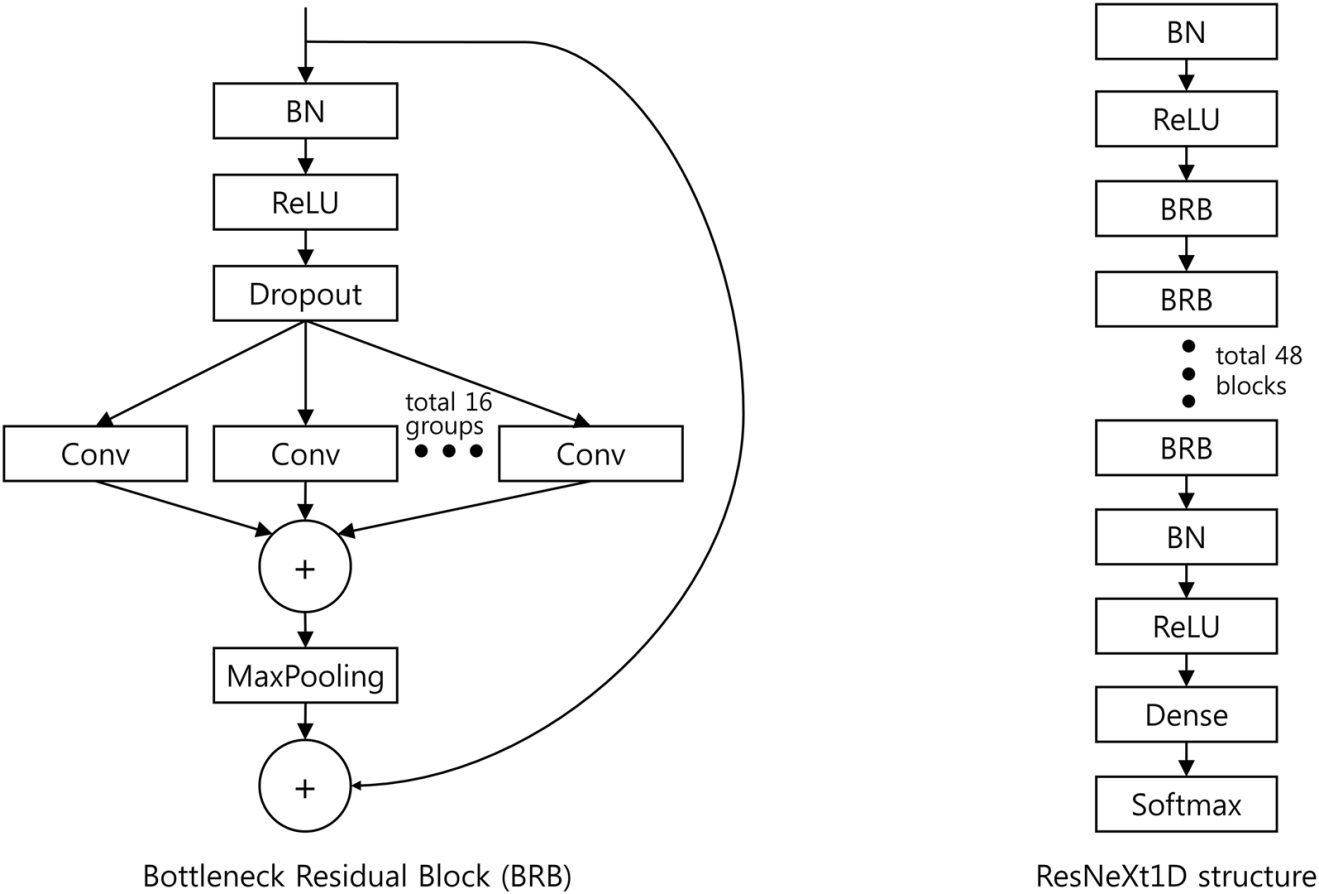
A bottleneck residual block (left) and our customized structure (right) of ResNeXt1D.

The Edi peaks detected using the LM method may include several false peaks owing to noise. To identify the true Edi peaks in the noisy signal, we applied the DNN. Since the Edi signal is continuous 1D data, we adopted ResNeXt1D^14^, which is useful for 1D signals, as the DNN backbone. Figure 3 shows the structure of ResNeXt1D customized for this study. For network learning, the training dataset consisted of detected peaks divided into true and false peaks based on the ground truth. The detected peaks from the LM are used as true peaks when the timestamp is within the detection range $\delta$ µs of the timestamp in the ground truth; otherwise, the detected peaks are false peaks. A fivefold cross-validation was used for the training, and testing sets were divided in an interpatient manner in the DNN classification. A random allocation of 80% of the training samples was used to train the DNN, and the remaining 20% was used for the validation.

### Respiration analysis

Because patient-ventilator asynchrony inevitably occurs during non-invasive ventilation, we evaluated double triggering and autotriggering events using our Edi peak detector method and compared it with the ground truth. Double triggering is an event in which two positive pressure ventilations are observed during one neural respiration by the Edi signal. Autotriggering is an event in which neural respiration is not observed in the Edi signal, but positive pressure ventilation exists. An episode of double triggering is defined as the period from the beginning of inspiration to the end of expiration in the Edi signal. An episode of autotriggering is defined as an event in which the interval time from the end of expiration to the beginning of the next inspiration of the Edi signal is longer than $t$.

From the beginning of inspiration to the end of expiration is identified as a respiratory event (RE), which is defined as

2 $$ RE\left( x \right) = \left\{ {\begin{array}{*{20}l}    {1,} \hfill & \begin{gathered}   if\;min\;Edi_{{a,b}}  = Edi(x),\;s.t.x \in \left[ {a,b} \right],x = \frac{{a + b}}{2}, \hfill \\   P_{k}  < a < b < P_{{k + 1}} ,Edi(x) < \sigma min(Edi(P_{k} ),Edi(P_{{k + 1}} )) \hfill \\  \end{gathered}  \hfill  \\    {0,} \hfill & {others} \hfill  \\   \end{array} } \right. $$

where ${P}_{k}$ is the timestamp of the $k$-th detected peak, and $\sigma$ is a scalar. Compared with Eq. (1), some constraints are added to Eq. (2), and the maximum operation is changed to the minimum operation. When searching for RE in the direction ${P}_{k+1}$ to ${P}_{k}$, the position of the first minimum Edi value searched is the beginning of inspiration. In contrast, when searching for RE in the direction ${P}_{k}$ to ${P}_{k+1}$, the position of the first minimum Edi value searched is at the end of expiration. The difference between $a$ and $b$ is the length ${\Omega }_{\mathrm{RE}}$ of the sub-signal. If the $RE\left(x\right)$ output is 1, timestamp $x$ is the beginning of inspiration or the end of expiration. The beginning of inspiration and the end of expiration were detected, and the respiration was identified using Eq. (2).

### Implementation details

We implemented ResNeXt1D used as a backbone network to predict the intensive care unit mortality^14^. In this study, the ‘kernel_size’, ‘stride’, ‘n_block’, ‘downsample_gap’, and ‘increasefilter_gap’ were set to 16, 2, 48, 6, and 12, respectively. Adam with a mini-batch size of 32 on one GPU, an initial learning rate of 0.001, and a weight decay of 0.001 was also used. To use ResNeXt1D, the ‘base_filters’ was set to 352. Because the Edi signal is a one-dimensional value and the final class is either a true or false peak, the input and output dimensions were set to 1 and 2, respectively. Other training setups followed the implementation of a previous study^15^. All the experiments were performed on a computer with GeForce RTX 3090 Ti, Python 3.7, and PyTorch 1.10.

### Tested algorithms and evaluation metrics

The performance of the proposed algorithm was compared with that of the three peak detection algorithms: the first derivative with an adaptive threshold (FDA)^16,17^, moving average with a dynamic threshold (MAD)^17^, and ServoTracker. The FDA splits the signal every 5 s and generates blocks. All signals were differentiated, and points with zero derivatives were extracted as peak candidates. The threshold was adaptively set according to the average amplitude at 2 s of the block. A peak with an amplitude greater than the threshold corresponds to the final peak. The MAD sets the signal to less than zero and then squares all signals. A block is created for the two moving averages, and the point with the largest amplitude value will ultimately be the peak point. ServoTracker was customized for the servo ventilators (Servo-i or Servo-n, Getinge, Gothenburg, Sweden).

Finding a peak within a single breath, consisting of inspiration and expiration, is vital for respiration analysis. Therefore, a detected peak was defined as a true positive when the timestamp of the detected peak was within a distance $\delta$ from the peak timestamp of the ground truth. The detected peak is a false positive if the distance between the detected peak and the ground truth is greater than $\delta$. The detected peak is a false negative if there is no detected peak within $\delta$ distance from the ground truth.

The following four evaluation metrics were used to compare the results of the proposed method with those of other methods for peak detection and asynchronous event detection performance

$$\mathrm{Precision}=\frac{\mathrm{TP}}{\mathrm{TP}+\mathrm{FP}}, \mathrm{Recall}=\frac{\mathrm{TP}}{\mathrm{TP}+\mathrm{FN}}, \mathrm{F}1-\mathrm{score}=2\frac{\mathrm{Precision}\times \mathrm{Recall}}{\mathrm{Precision}+\mathrm{Recall}},$$

$${\mathrm{R}}^{2}=1-\frac{\mathrm{SSE}}{\mathrm{SST}}, \mathrm{SSE}=\frac{1}{n}\sum_{i=1}^{n}{\left({y}^{\left(i\right)}-{\widehat{y}}^{\left(i\right)}\right)}^{2},\mathrm{SST}=\frac{1}{n}\sum_{i=1}^{n}{\left({y}^{\left(i\right)}-{\mu }_{y}\right)}^{2},$$

where, TP, FP, and FN are the true positive, false positive, and false negative, respectively. $\widehat{y}$ is the predicted value, and ${\mu }_{y}$ is the average of all ${y}^{\left(i\right)}$. The evaluation metrics precision, recall, and F1 score were implemented in Python 3.7. The Python module *sklearn.metrics.r2_score* was used to calculate ${\mathrm{R}}^{2}$.

## Results

Seventeen premature infants, born at a mean of 29^+6^ ± 1^+2^ weeks gestation with a mean birth weight of 1358 ± 358 g were included in the study. A mean of 54.7 ± 2.1 min of ventilator data were collected and used in the analysis.

In LM detection, we empirically determined the proper interval length ${\Omega }_{\mathrm{max}}$ to detect all true peaks while minimizing the number of detected false peaks. Therefore, ${\Omega }_{\mathrm{max}}$ and ${\Omega }_{\mathrm{min}}$ were set to 270 and 150, respectively. The number of residual blocks in ResNeXt1D was set to 48, and the scalar $\sigma$ in Eq. (2) was set to 0.5. The $\delta$ was set to 140, which was 10 ms less than the minimum distance between the peaks in the ground truth.

### Model training

**Figure 4:**
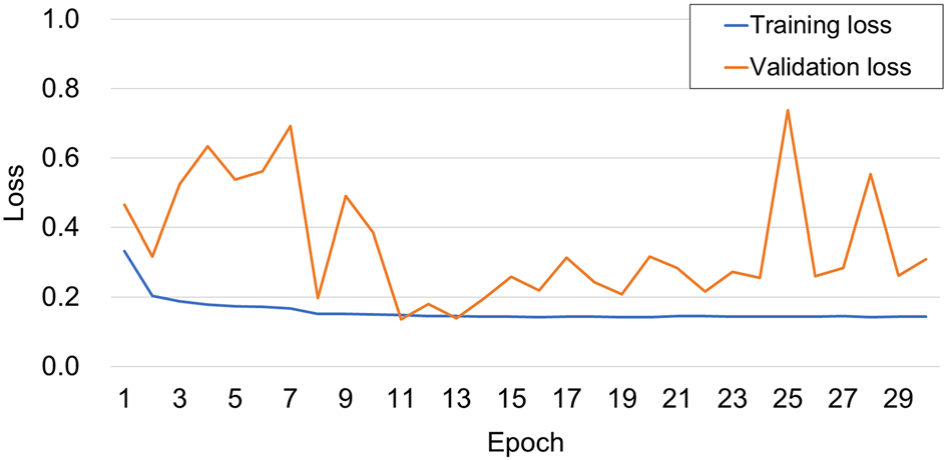
Training and validation losses for the number of epochs.

Using the learning scheduler *ReduceLROnPlateau*, the learning rate was reduced by 0.1 times when no loss improvement was observed for five epochs. The training was stopped when no loss improvement was observed for seven epochs. The weights with the minimum validation loss in the previous epochs were used for testing. Figure 4 shows that the training loss converges from the 12th epoch, the validation loss increases, and the model gradually overfits. Therefore, the model trained at 11 epochs was used for testing in the case of Fig. 4. The minimum validation losses in repeated experiments were generally observed within 20 epochs.

### Model complexity

We compared the model complexity of the proposed networks with four popular CNN architectures, as shown in Table 1. The number of trainable parameters (PN) and floating-point operations (FLOPs) are often used to measure model complexity related to space and time in neural networks^18,19^. We used both metrics because a model with fewer parameters may perform more calculations, or conversely, a model with more parameters may perform fewer calculations^19^. PN and FLOPs were measured by flops counter^20^. The proposed method has the largest model size (248 million parameters) but 2nd smallest FLOPs (12.44 × 10^8^).

**Table 1:** Comparisons of model size and complexity.

Model	FLOPs	PN (million)
AlexNet^21^	7.25 × 10^8^	58.3
VGG16^22^	1.55 × 10^10^	134.2
ResNet50^23^	3.80 × 10^9^	23.5
GoogLeNet^24^	1.57 × 10^9^	6.0
Proposed	12.44 × 10^8^	248.0

*FLOPs* floating point operations, *PN* the number of trainable parameters.

### Performance for Edi peak detection

The performances of FDA, MAD, ServoTracker, and our proposed method were compared for peak detection, as shown in Table 2. The proposed method was tested in two ways. The LM is the result of performing only LM detection without DNN classification. DNN is the result of performing the entire process of the proposed method, including LM detection and DNN classification. A representative fivefold cross-validation (CV) was performed for fair evaluation. A leave-one-out cross-validation (LOOCV) was also performed to determine the effect of patient-specific Edi signals on the evaluation performance owing to the small number of patients in the study.

**Table 2:** Edi peak detection performance. Values are presented as mean (standard deviation).

Method	Precision	Recall	F1-score	R^2^*
FDA	0.344 (0.092)	0.613 (0.211)	0.405 (0.084)	− 93.767 (86.661)
MAD	0.739 (0.201)	0.896 (0.232)	0.809 (0.213)	− 2.769 (5.404)
ServoTracker	0.614 (0.190)	0.993 (0.016)	0.742 (0.134)	− 22.133 (22.082)
LM (proposed)	0.620 (0.106)	**0.997** (**0.002**)	0.759 (0.087)	− 14.063 (12.731)
DNN, fivefold CV (proposed)	**0.970 (0.024)**	0.943 (0.027)	**0.956 (0.016)**	0.796 (0.200)
DNN, LOOCV (proposed)	0.968 (0.025)	0.946 (0.026)	**0.956 (0.016)**	**0.823** (**0.157**)

*Coefficient of determination of respiratory rate.

Significant values are in [bold].

*FDA* first derivative with adaptive threshold, *MAD* moving average with dynamic threshold, *LM* local maximum, *DNN* deep neural network with local maximum, *CV* cross validation, *LOOCV* leave-one-out cross-validation.

The proposed DNN method exhibited the best detection performance in the quantitative evaluation (Table 2). The LM method detected most of the true peaks while minimizing the number of detected false peaks. The peak detection of the ServoTracker was similar to the LM method. ServoTracker showed a high recall, but four of ten detected peaks were false peaks. MAD outperformed ServoTracker; however, the moving average had a limited ability to reduce the complex noise caused by motion. DNN exhibited the highest values in terms of precision and F1-score, while its standard deviations were the smallest. This indicates that the detector performance was the best and most stable. In calculating respiratory rates based on the number of Edi peaks, ${R}^{2}$ for respiratory rates of DNN was the highest, and its standard deviation was the smallest. The results of the proposed method for the fivefold CV and LOOCV were slightly different. The F1-scores were the same, but the precision and recall values were different. Because ${R}^{2}$ is sensitive to the peak detection, the results and corresponding standard deviations were larger than those of the detection metrics. The variation in the score was relatively large according to the performance change of the detection metrics.

**Figure 5:**
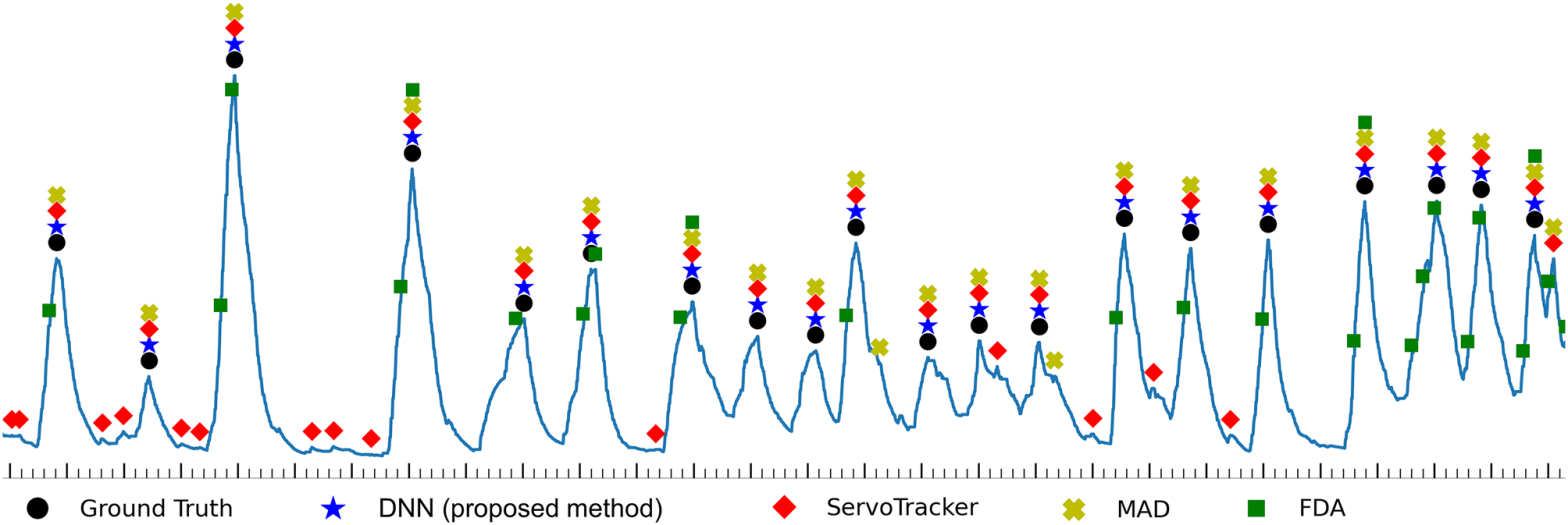
A representative Edi curve of peak detections by the ground truth, DNN (proposed method), ServoTracker, MAD, and FDA. The marker is a peak indication detected by each detector. The major and minor intervals on the x-axis are 1 and 0.2 s, respectively.

Figure 5 shows a representative Edi curve with peaks detected by the proposed method and other methods. The detected peak is a true peak when the x-axis position of the peak marker of a detector is equal to the ground truth marker (black circle); ServoTracker and FDA generally detected many false peaks. MAD detected some false peaks; however, the proposed method had the highest concordance with the ground truth.

### Asynchrony event analysis using detected Edi peaks

Table 3 shows raw data’s total number, precision, and recall values of asynchrony events for the peaks obtained by the comparison methods and the proposed method. The interval time $t$ related to autotriggering was set to 5 s. Because cases in which the interval from the end of expiration to the beginning of inspiration was over 5 s were fewer than 10 in each patient, the number of autotriggering was overall low. In the peak detection, MAD outperformed ServoTracker in F1-score and R^2^ but underperformed in asynchrony event detection. The proposed method showed that the number of double triggering and autotriggering was more similar to those of the ground truth than others. Because the proposed method accurately identified the peak, it also had the highest agreement rate with the ground truth for simultaneous events of mechanical ventilation and respiration. The 5-Fold CV and LOOCV results of the proposed method were similar to the peak detection results. The LOOCV results with relatively large training samples were closer to the ground truth.

**Table 3:** Detection of asynchrony events.

Method	Double triggering			Autotriggering		
	N (event/min)	Precision	Recall	N (event/h)	Precision	Recall
Ground truth	3.51	–	–	3.01	–	–
FDA	1.34	0.651	0.261	48.40	0.110	0.314
MAD	2.19	0.721	0.493	5.89	0.429	0.310
ServoTracker	2.49	0.843	0.614	1.23	0.250	0.286
DNN, fivefold CV (proposed)	3.65	0.874	**0.911**	3.27	0.596	**0.681**
DNN, LOOCV (proposed)	**3.57**	**0.886**	0.904	**3.01**	**0.692**	**0.681**

Significant values are in [bold].

*FDA* first derivative with adaptive threshold, *MAD* moving average with dynamic threshold, *DNN* deep neural network with local maximum, *CV* cross validation, *LOOCV* leave-one-out cross-validation.

## Discussion

Recently, there has been growing interest in mining vital sign data and building a big data registry from the data of hospitalized patients, especially those in intensive care units. However, any kind of vital sign data contains meaningless or fault data termed as noise which can significantly affect various data analysis and machine learning processes^25,26^. Handling of noise and obtaining relevant data from noisy vital signs should be performed to establish a clinically meaningful data registry^27^.

During ventilator care with NAVA, diaphragm electrical activity is monitored continuously and used to assess the level and pattern of respiration. Currently, the Edi signal is considered a respiratory vital sign that provides various pieces of information regarding breathing in patients with respiratory distress^4,7,8^. However, it is challenging to obtain reliable Edi signals, the electrical signals in the microvolt range, in an electrically noisy environment such as an intensive care unit with electrodes located in an electrically active esophagus, where peristalsis occurs, and next to the heart^28^. It is highly challenging to evaluate neural respiration using the obtained Edi signals, especially in premature infants with high respiratory rates and very small tidal volumes. Previous studies have reported double subtraction signal processing techniques, custom-designed filters, and algorithms for sensing disturbances to improve the quality of Edi signals^11,29,30^. However, the present study revealed that the currently used Edi analyzer (extracted from the ventilator using dedicated software, ServoTracker) had low precision in detecting the correct respiratory Edi peak. This resulted in a poor correlation in analyzing respiratory rates.

Complex and irregular noises negatively affect the performance of conventional analyzers. The signal may lose important information, such as delta, maximum, and minimum Edi, if the signal is modified to remove noise. For respiration analysis, we proposed a method to detect peaks in the original Edi signal without noise reduction using signal processing and deep learning. Traditional signal detection methods are based on CNNs and recurrent neural networks (RNNs)^31^. A 1D CNN^32^ is generally used; however, in continuous signals, many false detections can occur even if the detection accuracy of the model is high. Therefore, 1D CNN alone is not suitable to solve the problem. Long short-term memory^33^ and gated recurrent units^34^ based on RNNs face similar issues.

A two-stage approach that uses local maximal detection to over-detect candidate peaks and deep learning to classify candidates into final Edi peaks was proposed. In the first stage, the local maximum detection alone showed decent performance compared with other methods; some over-detected Edi peaks occurred in the first stage. Finally, most of the false peaks were eliminated by the 1D CNN. The proposed method exhibited high performance for continuous signals that detected many false peaks when directly applying the deep learning model. In addition, the proposed method outperformed other methods in terms of peak detection accuracy and double- and autotriggering similarities to the ground truth.

This study has two contributions: (1) respiration through the logical definition of events such as peak during neural inspiration, autotriggerings and double triggerings can be quantitatively analyzed, and (2) respiratory information in premature infants can be reliably monitored. Nevertheless, this study has some limitations. Because the input of the proposed method is one respiration consisting of the beginning of inspiration and the end of expiration, the proposed method cannot identify the Edi peak before the end of expiration. Therefore, this method cannot be directly applied to deliver respiratory assistance by the ventilator.

## Conclusions

We proposed a deep learning-based two-stage method to analyze the Edi of premature infants with a large amount of noise in the signal. The proposed method estimated the respiratory rates of premature infants more reliably by detecting Edi peaks more precisely. Because the proposed method showed promising results in all aspects of respiratory Edi peak detection performance in comparison with other methods, it could be used as a pre-processing step in building a respiration big data registry for analysis or machine learning processes.

In the future, this research can be extended to studies of (1) real-time Edi signal monitoring that provides information on the patient’s respiration, and (2) detection of inspiration beginning for breathing support during ventilator-assisted delivery through a manner similar to this study. These two additional studies based on the proposed method will help premature infants who require precise respiratory monitoring and assistance.

## Data Availability

The data supporting the findings of this study are available from the corresponding author upon reasonable request.
